# Interim Estimates of 2023–24 Seasonal Influenza Vaccine Effectiveness — United States

**DOI:** 10.15585/mmwr.mm7308a3

**Published:** 2024-02-29

**Authors:** Aaron M. Frutos, Ashley M. Price, Elizabeth Harker, Emily L. Reeves, Haris M. Ahmad, Vel Murugan, Emily T. Martin, Stacey House, Elie A. Saade, Richard K. Zimmerman, Manjusha Gaglani, Karen J. Wernli, Emmanuel B. Walter, Marian G. Michaels, Mary A. Staat, Geoffrey A. Weinberg, Rangaraj Selvarangan, Julie A. Boom, Eileen J. Klein, Natasha B. Halasa, Adit A. Ginde, Kevin W. Gibbs, Yuwei Zhu, Wesley H. Self, Sara Y. Tartof, Nicola P. Klein, Kristin Dascomb, Malini B. DeSilva, Zachary A. Weber, Duck-Hye Yang, Sarah W. Ball, Diya Surie, Jennifer DeCuir, Fatimah S. Dawood, Heidi L. Moline, Ariana P. Toepfer, Benjamin R. Clopper, Ruth Link-Gelles, Amanda B. Payne, Jessie R. Chung, Brendan Flannery, Nathaniel M. Lewis, Samantha M. Olson, Katherine Adams, Mark W. Tenforde, Shikha Garg, Lisa A. Grohskopf, Carrie Reed, Sascha Ellington, CDC Influenza Vaccine Effectiveness Collaborators, CDC Influenza Vaccine Effectiveness Collaborators, Adam S. Lauring, Julie Arndorfer, Daniel Bride, Ithan D. Peltan, Nicholas M. Mohr, David N. Hager, Matthew Prekker, Amira Mohamed, Nicholas Johnson, Jay Steingrub, Akram Khan, Laurence W. Busse, Abhijit Duggal, Jennifer G. Wilson, Nida Qadir, Christopher Mallow, Jennie H. Kwon, Matthew C. Exline, Nathan I. Shapiro, Cristie Columbus, Ivana A. Vaughan, Jarrod M. Mosier, Basmah Safdar, Estelle S. Harris, James D. Chappell, Laura S. Stewart, Sydney A. Swan, Pedro A. Piedra, Leila C. Sahni, Janet A. Englund, Danielle M. Zerr, Robert W. Hickey, John V. Williams, Chelsea Rohlfs, Elizabeth P. Schlaudecker, Dinah Dosdos, Mary E. Moffatt, Jennifer E. Schuster, Kirsten Weltmer, Peter G. Szilagyi, Tara Curley, Jamie Mills, Kiran Faryar, Robert A. Salata, Krissy Moehling Geffel, Mary Patricia Nowalk, Kempapura Murthy, Spencer Rose, Michael E. Smith, Brianna Wickersham, Brian D. Williamson, Natalie A.B. Bontrager, Olivia L. Williams, Joanna L. Kramer, Lora Nordstrom, Arnold S. Monto, Ivana A. Vaughn, Monica Dickerson, Callie McLean, Emma K. Noble, Caitlin Ray, Kelsey Sumner, Inih Essien, Linda Fletcher, Phillip Heaton, Sheryl Kane, Charlene McEvoy, Sunita Thapa, Gabriela Vazquez-Benitez, Cassandra Bezi, Richard Contreras, Gregg S. Davis, Bruno J. Lewin, Parag Mahale, Rudy Patrick, Lei Qian, Emily Rayens, Iris Anne C. Reyes, Denison S. Ryan, S. Bianca Salas, Lina S. Sy, Meiyu Yeh, Xi Zeng, Bruce Fireman, Kristin Goddard, John Hansen, Karen B. Jacobson, Julius Timbol, Ousseny Zerbo, Margaret Dunne, Yan Zhuang

**Affiliations:** ^1^Influenza Division, National Center for Immunization and Respiratory Diseases, CDC; ^2^Epidemic Intelligence Service, CDC; ^3^Biodesign Center for Personalized Diagnostics, Arizona State University, Tempe, Arizona; ^4^University of Michigan School of Public Health, Ann Arbor, Michigan; ^5^Washington University School of Medicine in St. Louis, St. Louis, Missouri; ^6^University Hospitals of Cleveland, Cleveland, Ohio; ^7^University of Pittsburgh School of Medicine, Pittsburgh, Pennsylvania; ^8^Baylor Scott & White Health, Temple, Texas; ^9^Baylor College of Medicine, Temple, Texas; ^10^Texas A&M University College of Medicine, Temple, Texas; ^11^Kaiser Permanente Washington Health Research Institute, Seattle, Washington; ^12^Kaiser Permanente Bernard J. Tyson School of Medicine, Pasadena, California; ^13^Duke Human Vaccine Institute, Duke University School of Medicine, Durham, North Carolina; ^14^UPMC Children’s Hospital of Pittsburgh, Pittsburgh, Pennsylvania; ^15^University of Cincinnati College of Medicine, Cincinnati, Ohio; ^16^Cincinnati Children’s Hospital Medical Center, Cincinnati, Ohio; ^17^University of Rochester School of Medicine and Dentistry, Rochester, New York; ^18^University of Missouri-Kansas City School of Medicine, Kansas City, Missouri; ^19^Children’s Mercy Hospital, Kansas City, Missouri; ^20^Baylor College of Medicine, Houston, Texas; ^21^Texas Children’s Hospital, Houston, Texas; ^22^Seattle Children’s Research Institute, Seattle, Washington; ^23^Vanderbilt University Medical Center, Nashville, Tennessee; ^24^University of Colorado School of Medicine, Aurora, Colorado; ^25^Wake Forest University School of Medicine, Winston-Salem, North Carolina; ^26^Kaiser Permanente Department of Research & Evaluation, Pasadena, California; ^27^Kaiser Permanente Vaccine Study Center, Kaiser Permanente Northern California Division of Research, Oakland, California; ^28^Division of Infectious Diseases and Clinical Epidemiology, Intermountain Health, Salt Lake City, Utah; ^29^HealthPartners Institute, Minneapolis, Minnesota; ^30^Westat, Rockville, Maryland; ^31^Coronavirus and Other Respiratory Viruses Division, National Center for Immunization and Respiratory Diseases, CDC.; University of Michigan; Intermountain Medical Center; Intermountain Medical Center; Intermountain Medical Center; University of Iowa; Johns Hopkins University; Hennepin County Medical Center; Montefiore Medical Center; University of Washington; Baystate Medical Center; Oregon Health and Science University; Emory University; Cleveland Clinic; Stanford University; University of California Los Angeles; University of Miami; Washington University in St. Louis; Ohio State University; Beth Israel Deaconess Medical Center; Baylor Scott & White Health and Texas A&M University School of Medicine; Henry Ford Health; University of Arizona; Yale University; University of Utah; Vanderbilt University Medical Center; Vanderbilt University Medical Center; Vanderbilt University Medical Center; Texas Children’s Hospital and Baylor College of Medicine; Texas Children’s Hospital and Baylor College of Medicine; Seattle Children’s Research Institute; Seattle Children’s Research Institute; UPMC Children’s Hospital of Pittsburgh and University of Pittsburgh School of Medicine; UPMC Children’s Hospital of Pittsburgh and University of Pittsburgh School of Medicine; Cincinnati Children’s Hospital Medical Center and University of Cincinnati College of Medicine; Cincinnati Children’s Hospital Medical Center and University of Cincinnati College of Medicine; Children’s Mercy Hospital and University of Missouri-Kansas City School of Medicine; Children’s Mercy Hospital and University of Missouri-Kansas City School of Medicine; Children’s Mercy Hospital and University of Missouri-Kansas City School of Medicine; Children’s Mercy Hospital and University of Missouri-Kansas City School of Medicine; University of Rochester School of Medicine and Dentistry; Washington University School of Medicine in St. Louis; Washington University School of Medicine in St. Louis; University Hospitals of Cleveland; University Hospitals of Cleveland; University of Pittsburgh School of Medicine; University of Pittsburgh School of Medicine; Baylor Scott & White Health; Baylor Scott & White Health; Baylor Scott & White Health; Kaiser Permanente Washington Health Research Institute; Kaiser Permanente Washington Health Research Institute; Duke Human Vaccine Institute; Duke Human Vaccine Institute; Phoenix Children’s Hospital; Valleywise Health Medical Center; University of Michigan School of Public Health; Henry Ford Health; CDC; CDC; CDC; CDC; CDC; HealthPartners; HealthPartners; HealthPartners; HealthPartners; HealthPartners; HealthPartners; HealthPartners; Kaiser Permanente Southern California; Kaiser Permanente Southern California; Kaiser Permanente Southern California; Kaiser Permanente Southern California; Kaiser Permanente Southern California; Kaiser Permanente Southern California; Kaiser Permanente Southern California; Kaiser Permanente Southern California; Kaiser Permanente Southern California; Kaiser Permanente Southern California; Kaiser Permanente Southern California; Kaiser Permanente Southern California; Kaiser Permanente Southern California; Kaiser Permanente Southern California; Kaiser Permanente Northern California; Kaiser Permanente Northern California; Kaiser Permanente Northern California;; Kaiser Permanente Northern California; Kaiser Permanente Northern California; Kaiser Permanente Northern California; Westat; Westat.

**Summary**

**What is already known about this topic?**

Influenza vaccines are reviewed biannually and updated as needed. In the United States, annual influenza vaccination is currently recommended for all persons aged ≥6 months.

**What is added by this report?**

Analysis of data from four vaccine effectiveness (VE) networks estimated interim pediatric influenza VE was 59%–67% in outpatient settings and 52%–61% against influenza-associated hospitalization. Interim adult influenza VE was 33%–49% in outpatient settings and 41%–44% against influenza-associated hospitalization.

**What are the implications for public health practice?**

These findings indicate that the 2023–24 seasonal influenza vaccine is effective at reducing the risk of influenza-associated outpatient visits and hospitalization. All eligible persons aged ≥6 months should receive annual influenza vaccination.

## Abstract

In the United States, annual influenza vaccination is recommended for all persons aged ≥6 months. Using data from four vaccine effectiveness (VE) networks during the 2023-24 influenza season, interim influenza VE was estimated among patients aged ≥6 months with acute respiratory illness-associated medical encounters using a test-negative case-control study design. Among children and adolescents aged 6 months–17 years, VE against influenza-associated outpatient visits ranged from 59% to 67% and against influenza-associated hospitalization ranged from 52% to 61%. Among adults aged ≥18 years, VE against influenza-associated outpatient visits ranged from 33% to 49% and against hospitalization from 41% to 44%. VE against influenza A ranged from 46% to 59% for children and adolescents and from 27% to 46% for adults across settings. VE against influenza B ranged from 64% to 89% for pediatric patients in outpatient settings and from 60% to 78% for all adults across settings. These findings demonstrate that the 2023–24 seasonal influenza vaccine is effective at reducing the risk for medically attended influenza virus infection. CDC recommends that all persons aged ≥6 months who have not yet been vaccinated this season get vaccinated while influenza circulates locally.

## Introduction

CDC's Advisory Committee on Immunization Practices recommends annual influenza vaccination for all persons aged ≥6 months (*1*). During previous influenza seasons, influenza vaccination prevented hundreds of thousands of outpatient medical visits, tens of thousands of hospitalizations, and thousands of deaths from influenza.* During the current influenza season, most influenza viruses detected were influenza A(H1N1)pdm09 viruses with cocirculation of influenza B/Victoria and influenza A(H3N2).^†^ Because circulating seasonal influenza viruses change continuously, influenza vaccines are reviewed biannually and updated as needed. CDC has monitored the effectiveness of annual influenza vaccines against circulating influenza strains since 2004.^§^ This report provides interim estimates of 2023–24 seasonal influenza vaccine effectiveness (VE) against laboratory-confirmed influenza for children, adolescents, and adults in the outpatient and inpatient settings from active and passive surveillance systems in 22 U.S. states.

## Methods

### Data Collection

Four analyses including patients who received medical care (outpatient or hospitalization) for acute respiratory illness (ARI) during the 2023–24^¶^ season were conducted using data from four CDC-affiliated VE networks: 1) Investigating Respiratory Viruses in the Acutely Ill (IVY), 2) New Vaccine Surveillance Network (NVSN), 3) U.S. Flu Vaccine Effectiveness (US Flu VE), and 4) Virtual SARS-CoV-2, Influenza, and Other respiratory viruses Network (VISION). These networks have been previously described (*2*–*5*).

IVY enrolled adult patients admitted to the hospital (Box). NVSN enrolled pediatric patients who received outpatient care** (outpatient clinics, urgent care, and emergency departments), and those admitted to the hospital. US Flu VE enrolled pediatric and adult patients who received outpatient care (outpatient clinics, urgent care, and emergency departments). VISION included pediatric and adult patients who received outpatient care (urgent care and emergency departments), and those admitted to the hospital.

BOX **Influenza vaccine effectiveness network characteristics — United States, 2023–2024 influenza season**

**IVY Network**

- **Population:** Adults aged ≥18 years
- **Settings**: No outpatient, inpatient only
- **Type of surveillance**: Active
- **Medical centers included (state)**: Baylor Scott & White Med. Ctr. – Temple (Texas), Baylor Scott & White Health, Baylor Univ. Med. Ctr. (Texas), Baystate Med. Ctr. (Massachusetts), Beth Israel Deaconess Med. Ctr. (Massachusetts), Cleveland Clinic (Ohio), Emory Univ. Med. Ctr. (Georgia), Hennepin County Med. Ctr. (Minnesota), Henry Ford Health (Michigan), Intermountain Med. Ctr. (Utah), Johns Hopkins Hospital (Maryland), Montefiore Med. Ctr. (New York), The Ohio State Univ. Wexner Med. Ctr. (Ohio), Oregon Health & Science Univ. Hospital (Oregon), Stanford Univ. Med. Ctr. (California), Ronald Regan UCLA Med. Ctr. (California), Univ. of Colorado Hospital (Colorado), Univ. of Iowa Hospitals (Iowa), Univ. of Miami Med. Ctr. (Florida), Univ. of Michigan Hospital (Michigan), Univ. of Washington (Washington), Vanderbilt Univ. Med. Ctr. (Tennessee), Wake Forest Univ. Baptist Med. Ctr. (North Carolina), Barnes-Jewish Hospital (Missouri), Univ. of Arizona Med. Ctr. (Arizona), and Yale Univ. (Connecticut)
- **Determination of vaccination status**: According primarily to vaccination registries or medical records (i.e., source documentation) and secondarily by self-report (if no source documentation available [<10% of cases])
- **ARI definition**: One or more of the following signs or symptoms: fever, cough, shortness of breath, new hypoxemia, or new pulmonary findings on chest imaging consistent with pneumonia

**NVSN**

- **Population:** Children and adolescents (aged 6 months–17 years)
- **Settings**: Inpatient and outpatient clinics, urgent care clinics, and EDs
- **Type of surveillance**: Active and passive
- **Medical centers included (state)**: Vanderbilt Univ. Med. Ctr. (Tennessee), Univ. of Rochester Med. Ctr. (New York), Cincinnati Children’s Hospital Med. Ctr. (Ohio), Texas Children’s Hospital (Texas), Seattle Children’s Hospital (Washington), Children’s Mercy Hospital (Missouri), and Children’s Hospital of Pittsburgh (Pennsylvania)
- **Determination of vaccination status**: State immunization registries, medical records or self-report.
- **ARI definition**: Signs and symptoms of acute respiratory illness (including cough, fever, or other symptoms) within 14 days of illness onset

**US Flu VE**

- **Population:** Children and adolescents aged 6 months–17 years; adults aged ≥18 years
- **Settings**: Outpatient clinics, urgent care clinics, and EDs; no inpatient
- **Type of surveillance**: Active
- **Medical centers included (state)**: Arizona State Univ. Tempe, Phoenix Children’s Hospital, Valleywise Health Med. Ctr. (Arizona), Univ. of Michigan and Henry Ford Health (Michigan), Washington Univ. in St. Louis (Missouri), Univ. Hospitals of Cleveland and Louis Stokes Cleveland Department of Veterans Affairs Med. Ctr. (Ohio), Univ. of Pittsburgh, Univ. of Pittsburgh Med. Ctr. (Pennsylvania), Baylor Scott & White Health – Temple (Texas), and Kaiser Permanente Washington (Washington)
- **Determination of vaccination status**: Medical records or state immunization registries and self-report (Michigan, Missouri, Ohio, Pennsylvania, Texas, and Washington sites); self-report only (Arizona site)
- **ARI definition**: Illness ≤7 days duration with new or worsening cough

**VISION**

- **Population:** Children and adolescents aged 6 months–17 years; adults aged ≥18 years
- **Inpatient versus outpatient settings**: Inpatient, urgent care clinics, and EDs
- **Type of surveillance**: Passive
- **Medical centers included (state)**: HealthPartners (Minnesota; and Wisconsin), Intermountain Health (Utah), Kaiser Permanente Northern California (California), Kaiser Permanente Southern California (California)
- **Determination of vaccination status**: Immunization information systems, electronic health records, claims data
- **ARI definition**: Acute respiratory clinical diagnoses or respiratory signs or symptoms based on ICD-10 codes

**Abbreviations:** ARI = acute respiratory illness; Ctr. = Center; ED = emergency department; ICD-10 = *International Classification of Diseases, Tenth Revision*; IVY = Investigating Respiratory Viruses in the Acutely Ill Network; Med. = Medical; NVSN = New Vaccine Surveillance Network; Univ. = University; US Flu VE = United States Influenza Vaccine Effectiveness Network; VISION = Virtual SARS-CoV-2, Influenza, and Other respiratory viruses Network.

### Data Analysis

Influenza VE was estimated based on a test-negative case-control design using multivariable logistic regression as (1 – adjusted odds ratio) × 100%. Case-patients were those with ARI who received a positive^††^ influenza molecular assay test result. Control patients were those with ARI who received a negative influenza molecular assay test result. Patients were considered vaccinated^§§^ if they had received ≥1 dose of 2023–24 influenza vaccine ≥14 days before an index date.^¶¶^ Patients were excluded*** if they were vaccinated within 13 days of the index date or received a positive SARS-CoV-2 test result (*6*). VE estimates were calculated for influenza A subtypes A(H1N1)pdm09 and A(H3N2) when possible. If more than one network had a VE estimate for the same age group, influenza type, and setting, VE was reported in the text as a range, from lowest VE point estimate to highest, without CIs.

Logistic regression models were adjusted for geographic region, age, calendar time of illness,^†††^ and other prespecified confounders.^§§§^ SAS software (version 9.4; SAS Institute) and R (version 4.3; R Foundation) were used to conduct the analyses. IVY, NVSN, and US Flu VE activities were reviewed by CDC, deemed not research, and were conducted consistent with applicable federal law and CDC policy.^¶¶¶^ VISION activities were reviewed and approved by the Kaiser Permanente Northern California, Kaiser Permanente Southern California, and Westat institutional review boards.****

## Results

### Vaccination Status Among Control Patients

During the 2023–24 influenza season, the proportion of patients with medically attended ARI who had received influenza vaccine varied by VE network, patient age, and setting. Among pediatric patients, the proportion of vaccinated control patients within the VE networks ranged from 25% to 31% in outpatient settings and from 32% to 41% in the inpatient setting. Among adult control patients aged 18–64 years, 28% to 37% in outpatient and 30% to 34% in inpatient settings were vaccinated; among control patients aged ≥65 years, 62%–68% in outpatient and 48%–60% in inpatient settings were vaccinated.

### Pediatric VE

VE against any influenza-associated ARI for children and adolescents aged 6 months–17 years ranged from 59% to 67% in outpatient settings and from 52% to 61% against any influenza-associated hospitalization (Table 1). VE against influenza A ranged from 46% to 59% in outpatient settings and from 46% to 56% against influenza-associated hospitalization. VE against influenza A(H1N1)pdm09 ranged from 54% to 61% in outpatient settings and against influenza-associated hospitalization was 60%. VE against A(H3N2) was 55% in outpatient settings. VE against influenza B ranged from 64% to 89% in outpatient settings.

**TABLE 1:** Number and percentage of children and adolescents aged 6 months–17 years receiving seasonal influenza vaccine, number and percentage with a positive or negative influenza test result, and vaccine effectiveness,* by influenza type^†^ and subtype^§^ — three networks, United States, 2023–24 influenza season

Network (setting)	Influenza test result by influenza vaccination status, no. vaccinated/No. total (%)		VE (95% CI)^¶^
	Positive	Negative	
**Any influenza**			
NVSN** (outpatient^††^)	123/622 (20)	793/2,577 (31)	59 (48–67)
US Flu VE (outpatient)	29/283 (10)	182/736 (25)	67 (48–80)
VISION (outpatient)	961/6,068 (16)	4,579/15,274 (30)	60 (57–64)
NVSN (inpatient)	29/128 (23)	543/1,321 (41)	61 (40–75)
VISION (inpatient)	21/113 (19)	299/921 (32)	52 (16–72)
**Any Influenza A**			
NVSN (outpatient)	84/411 (20)	793/2,577 (31)	55 (41–66)
US Flu VE (outpatient)	27/212 (13)	182/736 (25)	46 (15–67)
VISION (outpatient)	920/5,524 (17)	4,579/15,274 (30)	59 (55–62)
NVSN (inpatient)	25/102 (25)	543/1,321 (41)	56 (30–73)
VISION (inpatient)	21/105 (20)	299/921 (32)	46 (7–69)
**Influenza A(H1N1)pdm09**			
NVSN (outpatient)	61/298 (20)	793/2,577 (31)	54 (37–66)
US Flu VE (outpatient)	11/120 (9)	182/736 (25)	61 (26–81)
NVSN (inpatient)	18/79 (23)	543/1,321 (41)	60 (32–77)
**Influenza A(H3N2)**			
NVSN (outpatient)	19/87 (22)	793/2,577 (31)	55 (20–74)
US Flu VE (outpatient)	2/17 (12)	182/736 (25)	—
NVSN (inpatient)	4/10 (40)	543/1,321 (41)	—
**Influenza B**			
NVSN (outpatient)	39/216 (18)	793/2,577 (31)	64 (47–75)
US Flu VE (outpatient)	3/76 (4)	182/736 (25)	89 (70–97)
VISION (outpatient)	45/571 (8)	4,579/15,274 (30)	79 (71–85)
NVSN (inpatient)	4/27 (15)	543/1,321 (41)	—
VISION (inpatient)	0/10 (—)	299/921 (32)	—

**Abbreviations:** NVSN = New Vaccine Surveillance Network; OR = odds ratio; US Flu VE = U.S. Flu Vaccine Effectiveness network; VE = vaccine effectiveness; VISION = Virtual SARS-CoV-2, Influenza, and Other respiratory viruses Network.

* VE was estimated using the test-negative case-control design comparing vaccination odds among persons who had positive test results for influenza with vaccination odds among persons who had negative test results for any influenza and SARS-CoV-2. Calculated as (1 − adjusted OR) × 100%; ORs were estimated using logistic regression. Firth logistic regression was used for NVSN’s inpatient estimates.

^† ^Influenza A and B coinfections were included in both influenza A and influenza B VE estimates.

^§^ Influenza A subtype estimates were not calculated for VISION because of limited subtype data.

^¶^ All networks adjusted for geographic region, age, and calendar time. US Flu VE and VISION adjusted for sex and race and ethnicity. US Flu VE also adjusted for time since illness onset and self-reported health status. VE estimates with fewer than 50 cases or from models that did not converge are not presented and are indicated with a dash.

** Patients enrolled as outpatients in NVSN might have progressed to a more acute level of care, and those data might not be reflected in this analysis.

^††^ For NVSN and US Flu VE, outpatient setting is defined as outpatient clinics, urgent care, and emergency departments; for VISION, an outpatient setting is defined as urgent care and emergency departments.

### Adult VE

VE against any influenza-associated ARI for all adults aged ≥18 years ranged from 33% to 49% in outpatient settings and from 41% to 44% against any influenza–associated hospitalization (Table 2). VE against influenza A ranged from 27% to 46% in outpatient settings and from 40% to 42% against influenza-associated hospitalization. VE against influenza A(H1N1)pdm09 was 25% in outpatient settings and 50% against influenza-associated hospitalization. VE against influenza A(H3N2) was 54% in outpatient settings. VE against influenza B was 78% in two networks in outpatient settings and was 60% against influenza-associated hospitalization.

**TABLE 2:** Number and percentage of adults aged ≥18 years receiving seasonal influenza vaccine, number and percentage with a positive or negative influenza test result, and vaccine effectiveness,* by influenza type**^†^** and subtype^§^ — three networks, United States, 2023–24 influenza season

Network (setting)	Influenza test result by influenza vaccination status, no. vaccinated/No. total (%)		VE (95% CI)^¶^
	Positive	Negative	
**All adults (aged ≥18 years)**			
**Any influenza**			
US Flu VE (outpatient**)	177/568 (31)	803/1,807 (44)	33 (16–47)
VISION (outpatient)	4,501/18,385 (24)	21,356/52,657 (41)	49 (47–51)
IVY (inpatient)	200/632 (32)	1,517/3,872 (39)	44 (32–54)
VISION (inpatient)	728/1,839 (40)	7,425/14,168 (52)	41 (34–47)
**Any influenza A**			
US Flu VE (outpatient)	168/495 (34)	803/1,807 (44)	27 (9–43)
VISION (outpatient)	4,343/15,896 (27)	21,356/52,657 (41)	46 (44–48)
IVY (inpatient)	80/264 (30)	1,517/3,872 (39)	42 (23–57)
VISION (inpatient)	713/1,742 (41)	7,425/14,168 (52)	40 (33–47)
**Influenza A(H1N1)pdm09**			
US Flu VE (outpatient)	111/308 (36)	803/1,807 (44)	25 (1–43)
IVY (inpatient)	58/209 (28)	1,517/3,872 (39)	50 (30–64)
**Influenza A(H3N2)**			
US Flu VE (outpatient)	14/67 (21)	803/1,807 (44)	54 (11–77)
IVY (inpatient)	18/45 (40)	1,517/3,872 (39)	—
**Influenza B**			
US Flu VE (outpatient)	9/76 (12)	803/1,807 (44)	78 (57–90)
VISION (outpatient)	164/2,530 (6)	21,356/52,657 (41)	78 (74–81)
IVY (inpatient)	5/21 (24)	1,517/3,872 (39)	—
VISION (inpatient)	18/103 (17)	7,425/14,168 (52)	60 (30–77)
**Adults (aged 18–64 years)**			
**Any influenza**			
US Flu VE (outpatient)	136/489 (28)	503/1,368 (37)	25 (3–42)
VISION (outpatient)	2,557/14,698 (17)	9,194/33,086 (28)	52 (50–55)
IVY (inpatient)	87/383 (23)	579/1,927 (30)	49 (33–61)
VISION (inpatient)	197/773 (25)	1,367/4,050 (34)	40 (28–50)
**Any Influenza A**			
US Flu VE (outpatient)	128/417 (31)	503/1,368 (37)	13 (−13–34)
VISION (outpatient)	2,425/12,294 (20)	9,194/33,086 (28)	49 (46–51)
IVY (inpatient)	37/156 (24)	579/1,927 (30)	42 (13–61)
VISION (inpatient)	187/694 (27)	1,367/4,050 (34)	38 (24–48)
**Influenza B**			
US Flu VE (outpatient)	8/75 (11)	503/1,368 (37)	75 (50–89)
VISION (outpatient)	135/2,433 (6)	9,194/33,086 (28)	79 (75–82)
IVY (inpatient)	2/16 (13)	579/1,927 (30)	—
VISION (inpatient)	13/83 (16)	1,367/4,050 (34)	50 (5–74)
**Older adults (aged ≥65 years)**			
**Any influenza**			
US Flu VE (outpatient)	41/79 (52)	300/439 (68)	51 (14–72)
VISION (outpatient)	1,944/3,687 (53)	12,162/19,571 (62)	41 (36–45)
IVY (inpatient)	113/249 (45)	938/1,945 (48)	42 (23–56)
VISION (inpatient)	531/1,066 (50)	6,058/10,118 (60)	42 (34–50)
**Any influenza A**			
US Flu VE (outpatient)	40/78 (51)	300/439 (68)	52 (16–73)
VISION (outpatient)	1,918/3,602 (53)	12,162/19,571 (62)	40 (36–45)
IVY (inpatient)	43/108 (40)	938/1,945 (48)	47 (19–65)
VISION (inpatient)	526/1,048 (50)	6,058/10,118 (60)	42 (34–49)
**Influenza B**			
US Flu VE (outpatient)	1/1 (100)	300/439 (68)	—
VISION (outpatient)	29/97 (30)	12,162/19,571 (62)	69 (51–80)
IVY (inpatient)	3/5 (60)	938/1,945 (48)	—
VISION (inpatient)	5/20 (25)	6,058/10,118 (60)	—

**Abbreviations: **IVY = Investigating Respiratory Viruses in the Acutely Ill network; OR = odds ratio; US Flu VE = U.S. Flu Vaccine Effectiveness network; VE = vaccine effectiveness; VISION = VIrtual SARS-CoV-2, Influenza, and Other respiratory viruses Network.

* VE was estimated using the test-negative case-control design comparing vaccination odds among persons who had positive test results for influenza with vaccination odds among persons who had negative test results for any influenza and SARS-CoV-2. Calculated as (1 − adjusted OR) × 100%; ORs were estimated using logistic regression.

^†^ Influenza A and B coinfections were included in both influenza A and influenza B VE estimates.

^§^ Influenza A subtype estimates were not calculated for VISION because of limited subtype data.

^¶^ All networks adjusted for geographic region, age, and calendar time. IVY, US Flu VE, and VISION, adjusted for sex and race and ethnicity. US Flu VE also adjusted for time since illness onset and self-reported health status. VE estimates with fewer than 50 cases or from models that did not converge are not presented and are indicated with a dash.

** For US Flu VE, outpatient setting is defined as outpatient clinics, urgent care, and emergency departments; for VISION, an outpatient setting is defined as urgent care and emergency departments.

VE against any influenza-associated ARI for adults aged 18–64 years ranged from 25% to 52% in outpatient settings and from 40% to 49% against any influenza-associated hospitalization. VE against any influenza A ranged from 13% to 49% in outpatient settings and from 38% to 42% against influenza-associated hospitalization. VE against influenza B ranged from 75% to 79% in outpatient settings and was 50% against influenza-associated hospitalization.

VE against any influenza-associated ARI for adults aged ≥65 years ranged from 41% to 51% in outpatient settings and in two networks was 42% against any influenza-associated hospitalization. VE against influenza A ranged from 40% to 52% in outpatient settings and from 42% to 47% against influenza-associated hospitalization. VE against influenza B was 69% in outpatient settings. 

## Discussion

These interim estimates indicate that receipt of 2023–24 influenza vaccination reduced the risk for medically attended influenza-associated outpatient visits and hospitalization among children and adolescents and among adults, including those aged ≥65 years, consistent with results from previous years.^††††^ Influenza vaccination was effective against both influenza A (mostly subtype A(H1N1)pdm09) and B (lineage Victoria) viruses that have circulated so far this season, consistent with recent findings from Canada and Europe (*7**,**8*). VE estimates among adults ≥65 years, a group at increased risk for severe illness (*1*), were similar to those among adults aged 18–64 years. These findings support continuing efforts to increase influenza vaccination coverage to prevent influenza illness and associated hospitalization. Vaccination of persons aged ≥6 months who have not yet been vaccinated this season should continue while influenza viruses are circulating locally.

Influenza vaccination coverage in the United States has been lower this season than in the previous season and also lower than coverage before the COVID-19 pandemic.^§§§§^ In the current analyses, fewer than one half of test-negative control patients had received influenza vaccine in all VE networks and among enrollees of most age groups. The public health benefit of annual influenza vaccination depends on both vaccine effectiveness and vaccination coverage. Increased vaccination coverage will maximize prevention of influenza-associated illness, reducing both outpatient visits and hospitalization (*9*,*10*).

This is the first time interim pediatric and adult influenza VE estimates from four major networks have been presented together. Whereas previous interim VE estimates were for outpatient settings only, these analyses include estimates of VE among children and adolescents and among adults across a spectrum of illness severity. These findings are further strengthened by the geographic diversity of the networks, representing patients in 22 U.S. states.

### Limitations

The findings in this report are subject to at least four limitations. First, small sample sizes prevented estimation of VE for some age groups and settings. For example, an estimate of VE against influenza A(H3N2) was only possible in outpatient settings. Second, although models were adjusted for potential confounders, the potential for unmeasured confounding remained, such as underlying medical conditions or prior vaccination status. Third, there might be misclassification of vaccination status for networks that used self-reported vaccination data or if vaccine was administered outside of the medical system. Finally, in these analyses, patients who received ≥1 dose of 2023–24 influenza vaccine were considered vaccinated. However, to be considered fully vaccinated for the season, children aged 6 months–8 years are recommended to receive 2 influenza vaccine doses if they have not been previously vaccinated (*1*). Thus, some children classified as vaccinated might have only been partially vaccinated.

### Implications for Public Health Practice

Influenza vaccination remains the best way to prevent influenza. These findings provide further evidence of the importance of influenza vaccination in reducing medically attended influenza illness in outpatient and inpatient settings among all age groups. Last year alone, CDC estimates that influenza vaccination prevented about 6 million illnesses, 65,000 hospitalizations, and 3,700 deaths.^¶¶¶¶^ These findings support the recommendation for all persons aged ≥6 months to be vaccinated against influenza (*1*).

## Footnotes

* https://www.cdc.gov/flu/vaccines-work/past-burden-prevented-est.html

†
https://www.cdc.gov/flu/weekly/

§
https://www.cdc.gov/flu/vaccines-work/past-seasons-estimates.html

¶ Inclusion dates by network: IVY: September 1, 2023–January 31, 2024; NVSN: October 1, 2023–February 2, 2024; US Flu VE: October 10, 2023–January 24, 2024; VISION: October 15, 2023–January 15, 2024.

** Patients enrolled as outpatients in NVSN might have progressed to a more acute level of care, and those data might not be reflected in this analysis.

†† All influenza case-patients received a positive reverse transcription–polymerase chain reaction test result from a clinical or surveillance respiratory laboratory specimen for IVY, NVSN, and US Flu VE. For VISION, influenza case-patients received a positive molecular assay result from a clinical respiratory laboratory specimen.

§§ Vaccination status was self- or parent- or guardian-reported or abstracted from medical records, immunization information systems, or claims data.

¶¶ Index date for IVY, NVSN, and US Flu VE was date of ARI onset. Index date for VISION was the earlier of outpatient visit, hospital admission date, or influenza clinical testing date.

*** Patients with a positive SARS-CoV-2 test result were excluded in all networks because of the potential for bias from correlated vaccination probabilities. VISION participants with an influenza *International Classification of Diseases, Tenth Revision* (ICD-10) code but without a confirmatory laboratory test result or with COVID-19-related ICD-10 code even in the absence of a positive SARS-CoV-2 test were also excluded.

††† IVY used biweekly period of hospital admission. NVSN used month of patient enrollment in the hospital or outpatient setting. US Flu VE used month of illness onset. VISION used calendar date as a natural cubic spline.

§§§ IVY, US Flu VE, and VISION also adjusted for sex and race and ethnicity. US Flu VE also adjusted for days between illness onset and enrollment and self-reported general health status.

¶¶¶ 45 C.F.R. part 46.102(l)(2), 21 C.F.R. part 56; 42 U.S.C. Sect. 241(d); 5 U.S.C. Sect. 552a; 44 U.S.C. Sect. 3501 et seq.

**** 45 C.F.R. part 46.101(c); 21 C.F.R. part 56.

††††
https://www.cdc.gov/flu/vaccines-work/effectiveness-studies.htm

§§§§
https://emergency.cdc.gov/han/2023/han00503.asp

¶¶¶¶
https://www.cdc.gov/flu/about/burden-prevented/2022-2023.htm
